# A Tentative Model to Facilitate Intensive Care Professional Nurses’ Holistic Care in Private Hospitals in Gauteng, South Africa

**DOI:** 10.1177/08980101251334059

**Published:** 2025-06-25

**Authors:** Welhemina Molala, Charlene Downing

**Affiliations:** University of Johannesburg, South Africa

**Keywords:** model, facilitation, holistic, caring, self

## Abstract

**Introduction:** Holistic caring considers not only the physical aspects of a human being but also the interconnections among each individual's body, mind, and spirit. Holistic nursing focuses on the principles of holism: wellness and the interrelationship between human beings and their environment. Physical, emotional, mental, spiritual, and social well-being are essential to promote nurses’ ability to provide holistic care for patients. **Purpose of study:** The purpose of this research study was to develop, describe, evaluate, and provide guidelines for the operationalisation of a model to facilitate holistic self-care for professional nurses after the COVID-19 pandemic. **Design of study:** A theory-generating, qualitative, exploratory, descriptive and contextual research design was used. **Methods:** The researcher developed a model to facilitate intensive care professional nurses’ holistic care consisting of three phases, namely the relationship phase, the working phase and the termination phase. The model was evaluated and validated by five experts from five different universities in South Africa, using Chinn, Kramer's and Sitzmann framework. As an initial model grounded in qualitative insights, it requires further empirical testing, which will be pursued as part of postdoctoral research. **Findings:** It is anticipated that this study will enhance the sense of interconnectedness with oneself, others, and the environment at the physical, mental, emotional, and social levels. **Conclusions:** Nursing managers can facilitate nurses’ holistic self-care by providing resources and support, using this model as a frame of reference.

## Introduction and Background

Nursing recognises each person as a unique whole being and health as a state of well-being, not the absence of disease (Doe, 2022, p. 1). The available literature emphasises the importance of balancing physical, mental, emotional, spiritual, and social aspects of life in order to achieve overall well-being. Blaszko Helming et al. (2022, p. 99) allege that wholeness is frequently described as the harmony of body, mind, and spirit. Thus, wholeness must involve more than the intactness of physical structure and function or the status of isolated parts of a person. Ambushe, Awoke and Demissie (2023, p. 1) describe holistic nursing care as an approach to patient care that takes into account the physical, social, spiritual, and psychological needs of the patient. Nursing recognises each person as a unique whole being and health as a state of well-being, not the absence of disease (Doe, 2022, p. 1).

According to the American Holistic Nurses Association (AHNA), holistic care is a healing practice for the whole person. Mathew (2021, p. 381) proposed that holism refers to healing the whole person - body, mind and spirit, rather than solely focusing on the physical suffering and treatment of a disease or condition and that holistic care motivates the nurse to integrate self-care, self-responsibility, spirituality and reflection. Dykhuizen et al. (2022, p. 4) affirmed that one's health comprises several complex and overlapping aspects: mental, spiritual, physical and emotional.

Riegel et al. (2021, p. 2) echoed that Florence Nightingale's philosophy and teachings emphasised that the nurse must use her brain, heart and hands to create healing environments to care for the patient's body, mind and spirit. Florence Nightingale is considered to be the founder of holistic nursing. She taught nurses to focus on the principles of holism: unity, wellness and the interrelationship of human beings and their environment (Gripshi, 2021, p. 544). Muhammad-Lawal et al. (2023, p. 1) wrote that holistic nursing is based on the philosophy of holism and humanism and seeks to care for the whole person and find similarity in Ubuntu, which means humanity and respect for others in Nguni. Ubuntu is an African philosophy that provides communities with a holistic approach to care. However, the delivery of holistic care has become more complex in modern daily practice; owing to a shortage of resources, it is challenging for nurses to practice holistic self-care. Moreover, innovation programmes focus mainly on patient-centred care. Furthermore, patient-centred care programmes are becoming more prevalent.

The nursing profession faces numerous challenges, including technology and rapidly changing health policies. Ince et al. (2019, p. 2) reported that a chronic stress response mobilises leukocytes through sympathetic nerves and glucocorticoids; leukocytes secrete cytokines into the brain, inducing emotional changes. Microglia are also activated, leading to the activation of multiple neuro-immune pathways, resulting in emotional changes. When stress occurs, the neuro-endocrine system is activated, and microglia are activated, releasing hormones and neurotransmitters, increasing cortisol levels and suppressing the immune system (Alotiby, 2024, p. 2).

The literature confirms that, worldwide, workload increases during pandemics, leading to emotional exhaustion and poor concentration among healthcare professionals. A pandemic is a complex event involving a broad range of stressors that affect people's mental health (Taylor, 2022, p. 151). South Africa was also unprepared for the COVID-19 pandemic, as evidenced by overcrowded hospitals, a lack of resources and high mortality rates. A study by the same authors, “Lived *experiences of professional nurses caring for COVID-19 patients in private hospitals in Gauteng, South Africa: A phenomenological study*” (Molala & Downing, 2024), led to the development of this study. The aim of this study was to explore and describe professional nurses’ experiences caring for COVID-19 patients during the pandemic in private hospitals in Gauteng, South Africa. Three themes emerged: abrupt transition from normality to the COVID-19 pandemic, social isolation from one's family and community, lack of support from nursing management, and feelings of satisfaction and gratitude for teamwork and learning. A summary of the experiences of professional nurses caring for COVID-19 patients is presented in Figure 1.

**Figure 1.**
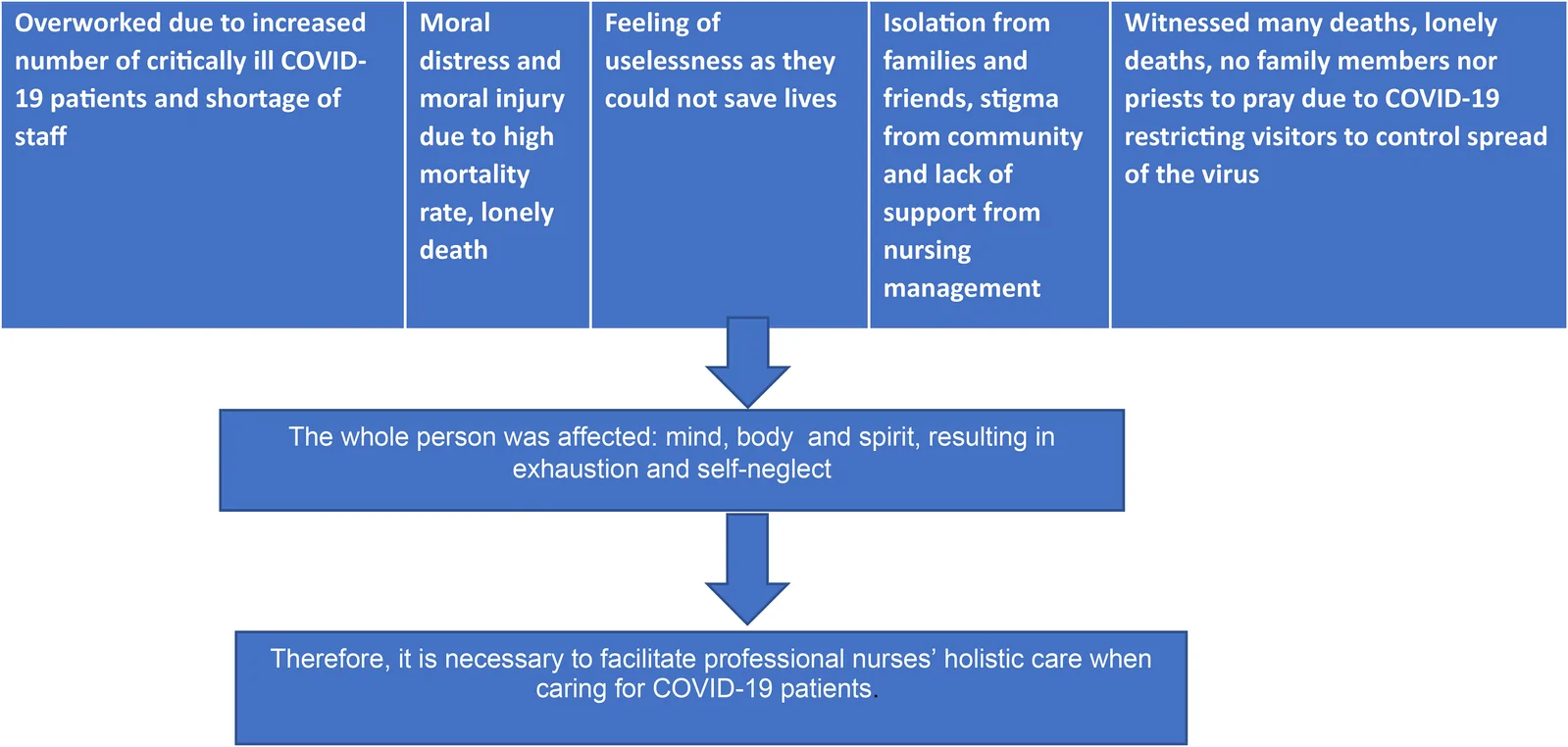
Summary of the experiences of professional nurses caring for COVID-19 patients.

## Study Design

A theory-generated, qualitative, exploratory, descriptive and contextual research design was used.

## Study Purpose

The purpose of this research study was to develop, describe, evaluate, and provide guidelines for the operationalisation of a model to facilitate holistic self-care for professional nurses post-COVID-19 pandemic

The researcher used five steps for generating the model (Chinn et al., 2022, pp. 148–157), as follows: Step 1, 1.1: Identification of central concepts, 1.2: Definition and classification of central concepts and the identification and classification of the central concept; Step 2: Relationship statements; Step 3: Development and description of the model; Step 4: Description of guidelines for the model for operationalisation; and Step 5: Evaluation of the model.

## Step 1: Concept Analysis

The concept ‘facilitation of holistic self’ was derived from Step 1.1 of concept analysis, using a qualitative, phenomenological, exploratory, descriptive, and contextual research design. The five largest private hospitals from the same hospital group in Gauteng were selected because they admitted many COVID-19 patients. Participants were selected through purposive sampling. Semi-structured, in-depth, individual interviews were conducted and audio-recorded field notes and observational notes were taken from April 2022 to December 2022. Data were saturated by the fifteenth participant. The interviews were transcribed verbatim and analysed using Giorgi's approach. **Results**: Three themes emerged: abrupt transition from normality to the COVID-19 pandemic; experienced isolation from family, community, and nursing management; and feelings of satisfaction and gratitude for teamwork and learning. An article was published thereafter: “*Lived experiences of intensive care professional nurses caring for COVID-19 patients in private hospitals in Gauteng, South Africa: A phenomenological study”* (Molala & Downing, 2024).

The researcher used English dictionaries, and subject and subject-related literature to define the central concepts. The literature was searched electronically via databases such as CINAHL, Medline, PsycINFO, ERIC, ScienceDirect, AMED, EBSCO (Health Source: Nursing and Academic Edition), Sage, UJoogle and Google Scholar. Concepts that were defined include facilitation, holistic, care and self.

*Facilitation is the act of assisting and simplifying a process, a dynamic and interactive function through which a facilitator fosters a positive environment by collaborating with the group* members through the mobilisation of resources.

*Holistic* implies dynamic and involves interaction and interconnection in healing and promoting health. Health is a relative state in which one can function well physically, mentally, emotionally, socially, and spiritually.

*Caring* may be defined as displaying kindness, helpfulness, affection and sympathy. Caring honours mind, body and soul and is a nurturing way of relating to and valuing others. It includes holistic components such as psychosocial, cultural and spiritual caring to promote health and growth.

*Self* is the totality of the individual and is about oneself and self-cognition, self-knowledge, self-reflection and self-evaluation. It is composed of a dynamic system that includes social, cultural, environmental and geographical structures, and learned attitudes and beliefs. The results of the concept analysis in Step 1.1 guided the model development process. The purpose of this study is to describe the development of a model for facilitating professional nurses’ holistic care as a frame of reference.

## Step 2: Relationship Statements

The relationship statements offer valuable insights into the complex dynamics of facilitating holistic self-care and emphasise the collaborative nature of the process. The assumptions of this model are based on Jean Watson's Theory of Human Caring. The core principle of this theory is the transpersonal caring relationship between the nurse and the patient, which goes beyond physical interaction to include spiritual and mental aspects.

The self-reflection and self-awareness of professional nurses with holistic self-care neglect were triggered by their negative lived experiences in caring for COVID-19 patients during the pandemic, and the social, psychological, spiritual, and emotional consequences needed to be addressed. The experienced professional nurse practising holistic self-care can assist professional nurses with holistic self-care deficits to reach their goal of healthy well-being and good quality of life by making the process easier.

Professional nurses with holistic self-care neglect are individuals in their own right, so it is essential to acknowledge that the facilitation of holistic self-care is dependent on their state of mind, body, and spirit. The professional nurse with holistic self-care neglect makes an autonomous choice to embark on the journey to holistic self-care. The experienced professional nurse practising holistic self-care understands that professional nurses with holistic self-care neglect do not exist in isolation but are part of a more comprehensive network of family, friends and community. The empowering journey towards holistic self-care involves a dynamic, interactive process and the mobilisation of resources for physical, mental, emotional, social, and spiritual well-being activities. This ongoing process nurtures the increasing confidence in and appreciation for holistic self-care. The experienced professional nurse practising holistic self-care skills facilitates and encourages the practice of holistic self-care as a life-long journey.

## Step 3: Description of the Development of the Model

This model provides the tools professional nurses with a holistic self-care deficit needed to practise holistic self-care during the COVID-19 pandemic. The model was developed according to the guidelines of Chinn et al. (2022, pp. 167–272). The model's structure is described as follows: the context of the model, the professional nurse with holistic self-care neglect, the experienced professional nurse practising holistic care, the facilitation process, and the dynamic interactive process of which the outcome is holistic self-care. The structure of this model is divided into three phases: the relationship phase, the working phase and the termination phase. The outcome is the achievement of practicing holistic self-care by the professional nurse with self-care neglect. Figure 2 below shows the structure of the model.

**Figure 2.**
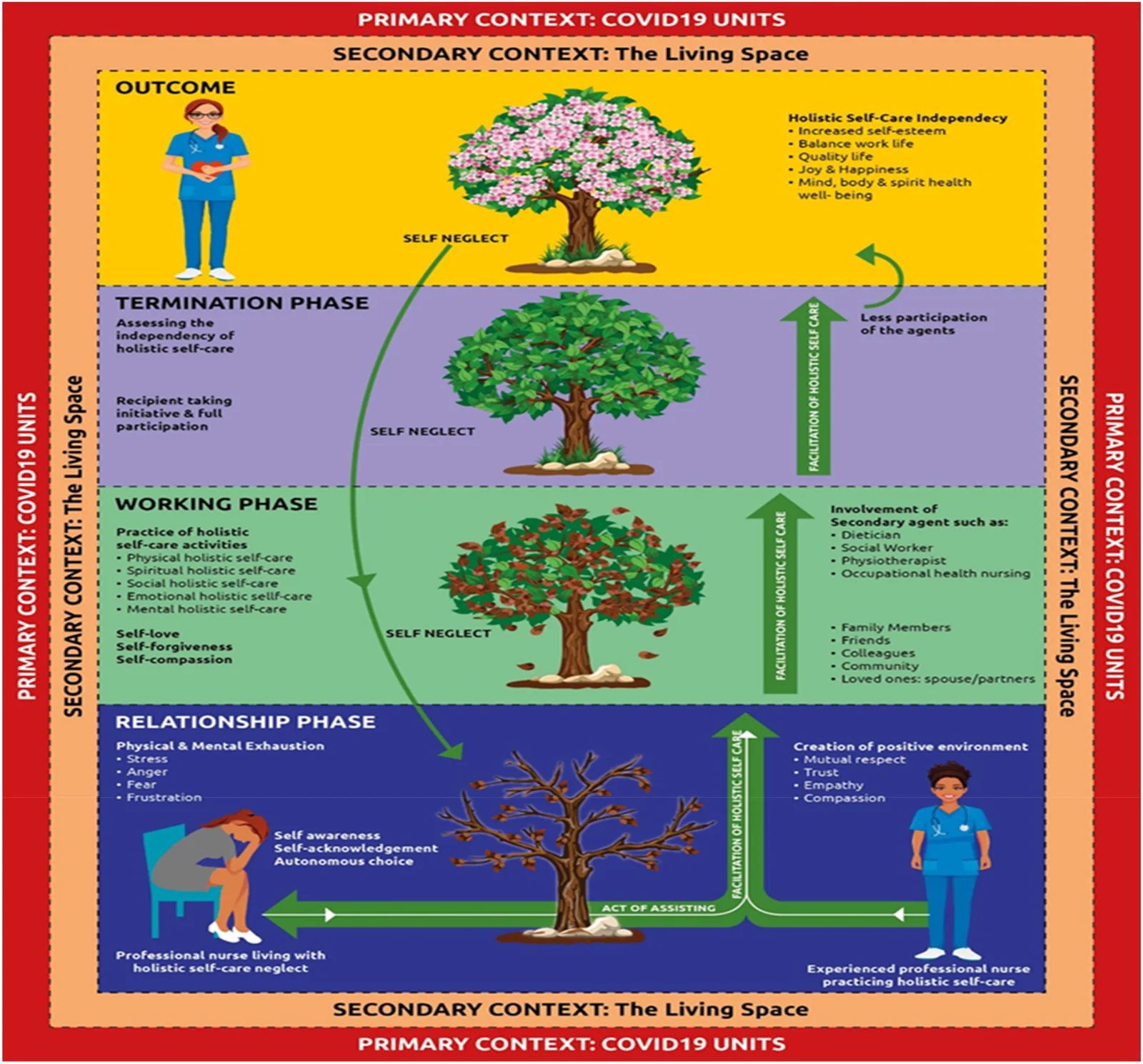
Structure of the model.

## Assumptions of the Model

The model's assumptions are explicitly linked to Jean Watson's Theory of Human Care (Watson & Woodward, 2010, p. 1), which emphasises the holistic nature of nursing care and the importance of nurses’ well-being in providing holistic self-care. This alignment provides a solid theoretical foundation essential for scholarly inquiry.

The assumptions are as follows:

Professional nurses’ health is more than the physical suffering and treatment of a disease or condition; they need to be facilitated in integrating self-reflection, self-awareness, self-care, and self-responsibility in their journey to life-long holistic self-care. NURSING ACT, 2005 (ACT No. 33 OF 2005) of South Africa, describe a professional nurse as a nurse who meets the prescribed educational requirements for registration as a professional nurse in the Regulations relating to the Approval of and the Minimum Requirements for the Education and Training of a Learner Leading to Registration in the Categories Professional Nurse and Midwife. According to Patricia Benner Novice to Expert Theory, professional nurse’ practice relies on written guidelines, procedures, and rules; depend on feedback from expert nurses is essential at this stage of nursing skill development (Ozdemir, 2019, p. 2).

The agent, who is an experienced professional nurse practising holistic self-care, is an expert nurse considered to offer assistance and care for a professional nurse with holistic self-care neglect through care, love, empathy, trust, understanding and self-reflection. Ozdemir (2019, p. 2) shared Benner Novice to Expert Theory, describing an expert nurse understands a situation and takes appropriate action based on their understanding rather than analytical principles. Nursing experts have an intuitive grasp of the situation and can identify the exact region of the problem without working through unproductive alternative diagnoses and solutions. Nikfarid et al. (2018, p. 4) concurred with Watson's theory and emphasised a caring and healing environment. They further stated that the internal and external factors that can help a person actualise his or her inner power of self-healing are called the environment, and a nurse is considered an external factor that can offer assistance and care to a person through the ten Caritas processes, including love, empathy, trust and teaching/learning experiences. Thus, the unity of the nurse and that person and the self-transcendence of both will occur in such enchanted moments.

The Canadian Holistic Nurses Association (2022, p. 6) noted that human beings are composed of internal and external environments, and that healing can occur at any level, including physical, cognitive, emotional, social, environmental and spiritual. The complexity of the whole-person system must be acknowledged, taking into consideration the ways in which experiences, relationships, community, culture, beliefs, values, and environment affect the health and well-being of the individual. Wei et al. (2021) stated that individuals can regain happiness and self-esteem through forgiveness, a process that increases self-esteem, positively affects their inner character, and helps them feel more satisfied and resilient. In all aspects of nursing activity, human values should be central, and the focus should be on respecting an individual's freedom, dignity, values, and beliefs (Ghanbari-Afra et al., 2022, p. 2). Neville et al. (2021) reported that nurses practise with compassion and respect to maintain the health, safety, and integrity of patients by promoting and protecting their rights, health, and well-being.

According to Gunawan et al. (2022, p. 3), three major concepts are required to achieve an interpersonal connection between two individuals: transpersonal caring dimensions, Caritas processes, and caring behaviour in a caring moment. The transpersonal caring dimensions refer to self-care and self-reflection, whereas the caritas processes pertain to the modalities or interventions the giver provides to the receiver, as well as acts of caring. Caring dimensions and Caritas processes encompass the behaviours the giver displays towards the receiver.

Human caring involves authentic presence, a deep belief in others, and the cultivation of one's spiritual practice toward wholeness of mind, body, and spirit. This type of care respects an individual's identity and values, supports the individual's uniqueness and independence, and helps people reach their maximum capacity (Ghanbari-Afra et al., 2022, p. 2). Fei (2020, p. 3) asserted that the self-identity of the body is central to body image; maintaining a coherent sense of self-identity requires embedding the body in daily interactions.

Spiritual well-being focuses on the relationship of professional nurses with God or any other spiritual beliefs. The physical, external environment includes both the primary context, the COVID-19 units where professional nurses with holistic self-care neglect work, and the secondary context, the living spaces where they stay. The social external environment involves interactions between families, friends, colleagues and members of the multidisciplinary teams in both the COVID-19 units and living spaces. The spiritual external environment refers to the cultural aspects, value systems and religious aspects of the professional nurses.

## The Structure of the Model

The concept of holistic care in nursing acknowledges the complexity of human beings and their interconnected physical, psychological, and social aspects (Soriano et al., 2019, p. 1). Jackson et al. (2023, p. 3) indicated that it is a basic human right for all individuals to be able to remain active, physically, mentally, socially, and spiritually, in a safe environment. Holistic care is a nursing approach that considers the whole person, assuming that biological, psychological, and social factors are interrelated (Sassen, 2023, p. 1).

The American Association of Colleges of Nursing (AACN, 2021) indicated that Florence Nightingale emphasised the influence of the environment on an individual's health and recovery as of the utmost importance. The concepts of health, healing, well-being, and interconnectedness with the multidimensional environment are reiterated in Florence Nightingale's work. Smith (2020, p. 9) named five concepts defining the nursing discipline as follows: human wholeness, health, healing and well-being, the environment-health relationship, and caring. The incorporation of a regime of self-care management enables nurses to achieve psychophysical balance and spiritual wellness. Nursing professionals will greatly benefit from setting aside time each day for holistic self-care practices that encourage self-reflection, freshen the spirit, and enable the continued administration of respectful, loving care to strengthen and revitalise their own physio-psycho-spiritual well-being (Nilsson, 2022, p. 64). Marsden and Cooper (2020, p. 2) indicated that it is vital for professionals to reflect on their knowledge and understanding of the person, including their physical, emotional and spiritual needs in facilitating and supporting the individual in addressing their holistic healthcare needs.

There is a partnership between professional nurses who neglect holistic self-care and experienced professional nurses practising holistic self-care. They engage collaboratively, share a common goal, and maintain a transpersonal relationship. Watson's theory (2007, p. 129) emphasises the significance of transpersonal teaching-learning-caring relationships, highlighting the importance of connecting with nurses on a deeply human level. This approach fosters trust, empathy, and mutual understanding, facilitating meaningful engagement. The experienced professional nurse practising holistic self-care acts to assist the professional nurse with holistic self-care neglect to embark on a life-long journey by creating an enabling environment that promotes the achievement of holistic self-care. This dynamic interactive process is terminated once the experienced professional nurse practising holistic self-care skills has assessed the professional nurses with holistic self-care neglect and finds that they are empowered and independent in practising holistic self-care.

The primary context was the ICU, where professional nurses faced negative experiences caring for COVID-19 patients during the pandemic. The secondary context was the living space where the professional nurse communicated with family, friends and colleagues. Khasoha et al. (2020, p. 4) reported that holistic nursing care is an important aspect of nursing practice in that it emphasises the care of the patient as a whole. In this study, the patient referred to the professional nurse with a holistic self-care deficit. The goals of holistic nursing are centred around improving health, reducing suffering, preventing illness, and protecting, promoting, and optimising health and wellness (Ambushe et al., 2023, p. 4). The experienced professional nurse practising holistic self-care facilitates the process that enables the professional nurse with holistic self-care neglect to participate in the lifelong journey that will enhance their holistic self-care. The experienced professional nurse practising holistic self-care mobilises the resources that will assist the professional nurses with holistic self-care neglect to take ownership of holistic self-care. In this model, the experienced professional nurse practising holistic self-care and the secondary agents are the resources that facilitate the professional nurses’ holistic self-care. The secondary agents include social workers, counsellors, occupational health nurses, dieticians and physiotherapists, colleagues, family, friends and the community.

The process of facilitating professional nurses’ holistic care occurs in three phases, namely the relationship phase, the working phase, and the termination phase.

## Relationship Phase

The relationship phase is the first phase in the process of facilitating holistic self-care. The colour blue was used; blue, as a colour, was interpreted by Kauppinen-Räisänen and Jauffret (2018, p. 2) as a cure and can be explained by its iconic relation to objects such as water and the sky, which may, in specific geographical contexts, be perceived as not only cold but also fresh and calming. This is beneficial to mind, body and soul. Blue can be considered an indexical sign representing a cure or a fresh and calming impact on the body. A voluntary act of assisting begins with the green arrow starting from the experienced professional nurse practising holistic self-care, pointing to the professional nurse with holistic self-care neglect. This shows that the experienced professional nurse practising holistic self-care is willing to voluntarily assist the professional nurse with holistic self-care neglect. The colour green was used for this arrow. Keidel et al. (2019, p. 2) stated that green is associated with starting or moving forward. The two white arrows within the green arrows point from each side and merge into a single upwards arrow, indicating collaboration and transpersonal relationships. The white arrows indicate purity and transparency. The colour white symbolises purity and has a positive connotation (Kemertelidze & Giorgadze, 2021, p. 1). In this model, white arrows indicate clear communication, openness, and a commitment to transparency in the facilitation process.

Khawaja et al. (2022, p. 2) alleged that the tree symbolises the interconnected nature of all life. This implies that all the tree's parts are connected and part of a whole. In this study, a tree was used as a metaphor to present an individual. Similarly, the body, mind and spirit form a complete whole. The leaves represent observable manifestations of health. Healthy leaves demonstrate beauty and physical, mental, emotional, social and spiritual health. In the relationship phase, the tree has a few leaves that change colours to brownish instead of greenish; this is a sign that the professional nurse with a holistic self-care deficit is not healthy.

## The Working Phase

This is the most significant phase, which is green in colour; this is where most activities take place. Haller (2019, p. 2) described the colour green as follows: it is often associated with balance and stability, evokes a sense of tranquillity, peace, and harmony, represents new beginnings, and is the colour of life and growth. Green is the colour of grass, leaves and vegetables and naturally suggests rest, cool shade and refreshment, all of which are pleasant aspects (Vijaya, 2023, p. 3). In this model, the professional nurse with holistic self-care neglect is assisted and empowered in the journey to healthy well-being and a good quality of life; this is represented by green, healthy leaves. Green is associated with peace, trust, cleanliness, and enthusiasm (Demir, 2020, p. 1).

Professional nurses with holistic self-care neglect are self-innovated and willing to participate in all activities that enhance their health and well-being. The senior nurse manager who possesses holistic self-care skills mobilises the resources; various activities by various secondary agents take place in the working phase: physiotherapists, dieticians, social workers, dieticians, and occupational healthcare nurses. During the process, there is a probability of self-neglect. Self-neglect is a behavioural condition characterised by a neglect of basic needs such as personal hygiene, clothing, feeding, or caring for medical conditions according to their needs. Alternatives can be explored if fear of change is an issue. Explaining that there are alternative ways forward may encourage the person to get involved. Self-neglect in the context of this model refers to regression or deterioration of the progress made by professional nurses with holistic self-care neglect during the facilitation process. It represents a setback or a return to previous levels of holistic self-care neglect after initial progress or improvement has been made. In the working phase of the model, self-neglect is indicated by the branches of the metaphorical tree becoming dry and the leaves turning brownish and falling down.

## The Termination Phase

This is the end phase, the end of the journey between the experienced professional nurse practising holistic self-care skills and the professional nurse with holistic self-care neglect. The colour purple was used as it was described by Anwer (2023) that it is associated with a variety of meanings, including wisdom, creativity, royalty, power, ambition, and luxury. It can also represent magic, extravagance, peace, pride, independence, and wealth. In the termination phase, the professional nurses with holistic self-care neglect are at peace, independent, and able to practise holistic self-care by themselves. Purple is often associated with creativity, wisdom, and spirituality. It is believed to inspire high ideals and enhance the imagination. Purple has a calming effect on the mind, reducing stress and other emotional disturbances. Purple is capable of inducing calmness and happiness. In addition, the colour purple is said to be able to stimulate intuition and creativity. Being detached from physical, corporal and earthly logic in general, purple opens the gates to supernatural and transcendent worlds that can be specific yet elusive at the same time (Daugaard et al., 2022). The experienced professional nurse practising holistic self-care evaluates the professional nurse with holistic self-care neglect, and their abilities to achieve holistic self-care independence. Once the desired outcome has been achieved, termination occurs. In this phase, the professional nurses with holistic self-care neglect are skilled in holistic self-care.

## The Outcome

The outcome is represented by the symbol of a bright yellow flowering tree which signifies that the professional nurses with holistic self-care neglect can practise holistic self-care. Yellow is associated with cheerfulness, joy and happiness. Finally, yellow flowers represent specific successes that professional nurses with holistic self-care neglect value and want to share and celebrate the lifelong journey of holistic self-care. Figure 3 depicts a graphical representation of the outcome.

**Figure 3.**
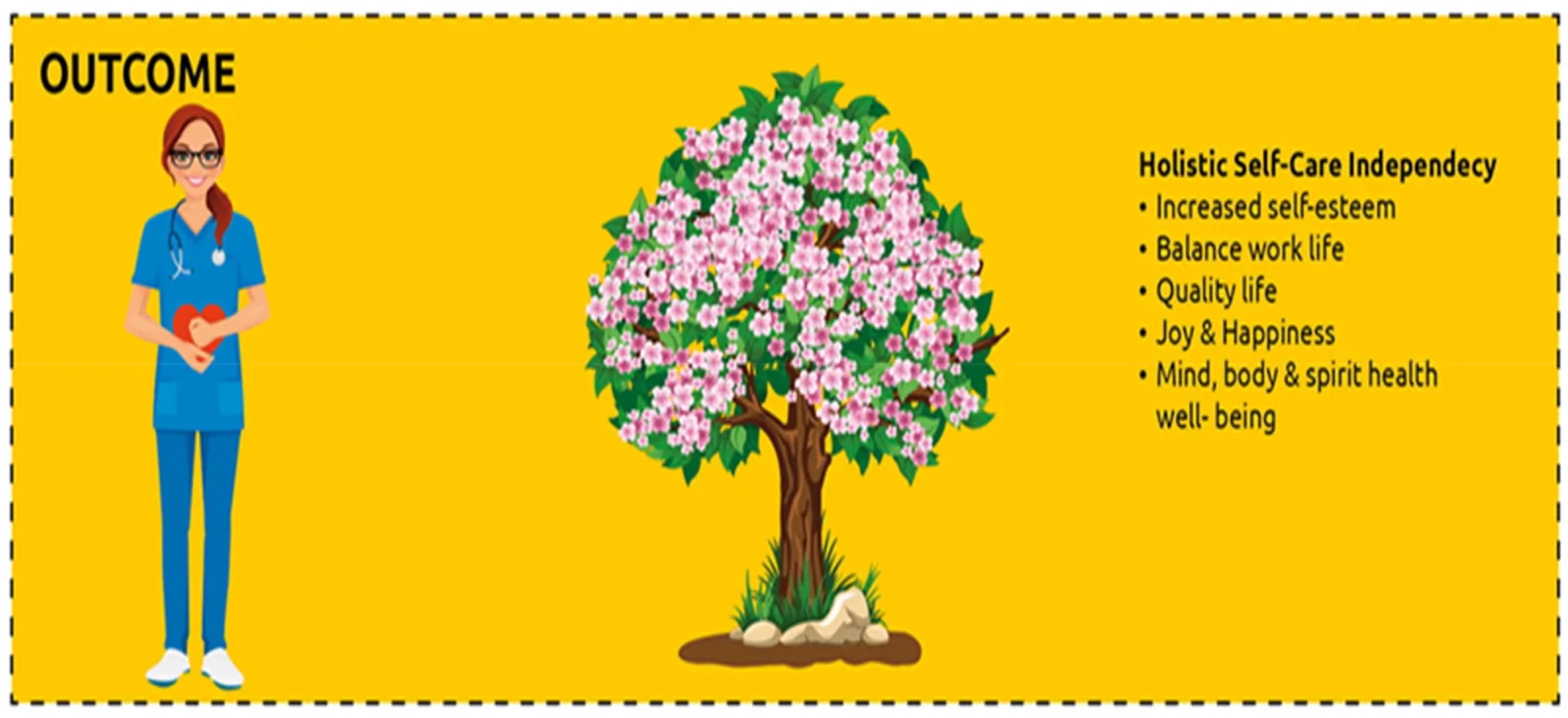
Illustration of the outcome.

## Evaluation of the Model

The evaluation and validation of the model were conducted by experts in model development, utilizing Chinn, Kramer's and Sitzman framework. Full-scale model testing has not yet been conducted; however, this will be pursued as part of postdoctoral research. Doctoral TEAMS was scheduled to evaluate the model. Five experts from five different universities in South Africa attended. All the evaluators have more than five years of experience in qualitative research and theory generation. The presentation, along with questions and comments, took 60 min. The experts provided immediate verbal feedback, and later the completed evaluation forms were received by email. A summary of the demographic data of the experts who evaluated the model is presented in Table 1 below.

**Table 1.** Demographics of the Research Panel Experts

Evaluator	Highest qualification	Current job title	Years of experience in qualitative research	Years of experience in theory generation
1	PhD in Nursing Science	Professor	24 years	5 years
2	PhD in Nursing Science	Lecturer and researcher	10 years	10 years
3	PhD in Nursing Science	Associate professor	7 years	6 years
4	DLitt et Phil	Associate professor	11 years	5 years
5	DCur	Lecturer	11 Years	6 years

The criteria set forth by Chinn et al. (2022, pp. 169–180) were used as follows:

- How clear is the model?
- How simple is the model?
- How general is the model?
- How accessible is the model?
- How important is the model?

## Clarity

Chinn, Kramer and Sitzman's criteria (2022, p. 160) were followed to address the clarity and consistency of the concepts and statements within the model; it was about understanding the model as well as conceptualising the ideas consistently. The concept of clarity refers to the way definitions enhance an understanding of the model, and concepts within the model must be consistent with their definitions (Chinn et al., 2022, p. 203). The research supervisor provided guidance and corrections where needed to ensure that the model was clear. The model evaluators found it to be clear and simple to follow and described the model's clarity in the following ways:

Evaluator 1: “Very clear, Ms Molala provided a very clear explanation of the model, well-structured and complete.”

Evaluator 2: “Very clear.”

Evaluator 3:” Very clear.”

Evaluator 4: “Moderately clear. Some arrows are labelled whilst some are not, e.g., working and termination phase.”

Evaluator 5: “Moderately clear.”

## Simplicity

A simple design allows descriptive concepts and interactions to be kept to a minimum. Concepts were kept simple, and the researcher avoided overly complex descriptions.

Evaluator 1: “Very simple. The model is easy to understand.”

Evaluator 2: “Very simple. The arrows and presentation of the tree are very clear.”

Evaluator 3: “Moderately simple

Evaluator 4: “Moderately simple; once all arrows are labelled, it will be better.”

Evaluator 5: “Extremely simple.”

## Generality

According to Chinn et al. (2022, p. 208), generality refers to the extent to which the model covers a wide range of issues and purposes that can be applied to other settings or nursing situations.

Evaluator 1: “Very general. The model is applicable in COVID-19 units, but also wider, in different units where nurses are working, because similar circumstances exist there.”

Evaluator 2: “Very general. The model can be used outside COVID-19 units, but possibly within any clinical healthcare context.”

Evaluator 3: “Very general.”

Evaluator 4: “Very general; addresses COVID 19 setting in general.”

Evaluator 5: “Extremely general.”

## Accessibility

The accessibility of a model is the extent to which empirical indicators can be identified for its concepts. A key component of this evaluation is determining if the model is meeting its purpose and whether the experience can be applied to practice in order to evaluate its effectiveness.

Evaluator 1: “Very accessible. Based on the fact that the model is clear, simple and general, it is highly accessible.”

Evaluator 2: “Very accessible.”

Evaluator 3: “Very accessible.”

Evaluator 4: “Very accessible, with more clear labelling of the model components, it will get better.”

Evaluator 5: “Extremely accessible. There is empirical evidence. It shows the main themes, relationships and attributes.”

## Importance

The degree to which a theory leads to useful goals in practice, research, and education. Chinn et al. (2022, p. 210) argued that a theory or model's importance is determined by its clinical relevance.

Evaluator 1: “Extremely important. Well done on developing a model for this very important topic. I have much appreciation for the concept of ‘holistic self-care neglect’, I think it captures the nurses’ experiences very well. The impression I got from the themes Ms Molala shared in her presentation was that nurses working in COVID-19 wards experienced being fragmented in their internal and external environment, and therefore, the symbol of a tree as a reflection of the interconnectedness of life and recovering this interconnectedness, is relevant and beautiful.”

Evaluator 2: “Extremely important. As a caring profession, it is often difficult to self-care. Self-care is important to be able to care for nurses.”

Evaluator 3: “Very important”

Evaluator 4: “Very important. The model is very important; we need models to help with managing self during pandemics.”

Evaluator 5: “Extremely important.”

## Contribution

This study could increase awareness among healthcare organisations that the total well-being (mind, body and spirit) of nurses and all other healthcare providers is paramount to delivering quality patient care. In turn, this will help to decrease burnout and may increase nurse retention. This study could drive healthcare institutions to plan for future pandemics and respond to nurses’ health, care and support needs, as South Africa does not have clinical resource organisations that support nurses’ holistic self-care, such as the American Holistic Nurses Association (AHNA), which supports the well-being of nurses. It is anticipated that this study will enhance the sense of interconnectedness with oneself, others, and the environment at the physical, mental, emotional, and social levels. This model can be used as a frame of reference for any other pandemic by healthcare workers and even any other profession not related to health care.

This model is relevant to nursing education, student nurses, nursing research and nursing practice, and empowers individuals to love or mentor others.

## Limitations of the Study

### Sample Limitation

Fifteen nurses were recruited from five private hospitals, for this study, but none from state hospitals. Therefore, the findings cannot be generalised to other populations. Nursing categories such as enrolled and auxiliary nurses were excluded, limiting the overview of nurses’ experiences during the COVID-19 pandemic. The qualitative nature limits generalisability, which could be addressed in future studies by using a quantitative method with a larger sample size.

### Temporal Limitation

The study was conducted from the end of the fourth wave to the end of the fifth wave of the pandemic; if it had been conducted during the first wave, more experiences might have been shared as it happened.

### Research Bias

The first author is a deputy nurse manager, and although the study was not conducted in the hospital where she was employed, it was within the same hospital group; thus, participants might not have felt comfortable sharing some of their experiences.

## Conclusions

The results revealed that professional nurses operated in stressful work environments during the COVID-19 pandemic, leading to feelings of overwhelm and exhaustion across physical, mental, emotional and spiritual dimensions. This study highlighted various sources of stress in the working environment that negatively affect nurses’ overall well-being. The extra demand for patient care resulted in self-care neglect. Improving the well-being of professional nurses will, therefore, require multilevel and multifaceted approaches that address their holistic needs: mind, body and spirit. Creating safe, supportive environments is essential for the well-being of professional nurses. An individual's well-being is interconnected; neglecting one aspect will compromise other aspects. It is vital that nurses are healthy physically, mentally, emotionally, socially and spiritually in order to effectively meet their complex needs, as well as those of their patients and their communities. Nurses and other healthcare practitioners can benefit from guidelines for implementing holistic self-care during any pandemic. The model can benefit nursing practice, nursing research, and nursing education. By using this model as a frame of reference, nursing managers can facilitate nurses’ holistic self-care by providing resources and support.

## Recommendations

- **Nursing practice**

The model can be utilised as a frame of reference by other healthcare professionals such as radiographers, physiotherapists, social workers and doctors to facilitate holistic self-care of other healthcare workers. The ICU is a strenuous environment due to the complexity of patients’ diagnoses and the equipment. This model will guide ICU nurses to apply learned skills and coping mechanisms to their daily lives in expressing their emotions and addressing any fears they might have.

- **Nursing research**

More nursing research is needed that emphasises holistic self-care and collaborative efforts between nurses and community groups, health agencies, and support groups. Studies on holistic self-care could be conducted in public health hospitals. Further research is needed to implement a model to facilitate professional nurses’ holistic care.

- **Nursing education**

As a curriculum integration, the use of this holistic self-care model will empower nurse educators to teach and transfer theoretical knowledge of holistic care into clinical practice. Nursing students’ confidence will increase, and they will be able to provide holistic care to patients. Thus, in turn, student nurses will be able to provide education to the patients regarding holistic self-care practices and promote healthy lifestyles that can improve their overall health. Patients will be encouraged to participate actively in their own healing process. The model should be integrated into both undergraduate and postgraduate training programmes.

- **Future research**

Research should be conducted to establish whether integrating the model into nursing practice and the nursing curriculum can benefit nursing students in their ability to cope effectively.

## Ethical Considerations

The study was approved by the Faculty of Health Sciences of the UJ Higher Degrees Committee (HDC-01-96-2021), the UJ Faculty of Health Sciences Ethics Committee (Rec-1252-2021) as well as the private hospital group, the Research Operations Committee (UNIV-2021-0054).

## Consent to Participate

The principle of respect for autonomy considers self-determination as the basis of informed written consent was followed in the published study that led to this study, by the same authors, “Lived experiences of professional nurses caring for COVID-19 patients in private hospitals in Gauteng, South Africa: A phenomenological study (Molala & Downing, 2024) as below:

The researcher met with the management of each participating private hospital on separate days. In each case, the management delegated the deputy nurse managers, as they handled more operational nursing duties and regularly interacted with the nurses in their daily routines. It was the researcher's responsibility to ensure that the professional nurses were not coerced by the gatekeeper to take part in the study as outlined follows: The informed consent forms and the letter for participation were distributed to the participants by the gatekeepers. However, each participant provided their own signed informed consent form to the researcher to ensure no- coercion informed consent by gatekeepers The participants signed an informed consent form for audio recording as well. Identifiable information was removed, such as the names of the private hospitals, and the names of the participants were replaced by the numbers.
